# Severe Refractory Immune Thrombocytopenia Successfully Treated with High-Dose Pulse Cyclophosphamide and Eltrombopag

**DOI:** 10.1155/2015/583451

**Published:** 2015-06-09

**Authors:** Faiz Anwer, Seongseok Yun, Anju Nair, Yusuf Ahmad, Ravitharan Krishnadashan, H. Joachim Deeg

**Affiliations:** ^1^Department of Medicine, University of Arizona, Tucson, AZ 86721, USA; ^2^Medical Oncology, Arizona Oncology Associates, Tucson, AZ 85704, USA; ^3^Fred Hutchinson Cancer Research Center and the University of Washington, Seattle, WA 98109, USA

## Abstract

Severe refractory ITP is clinically challenging and a variety of single or combination chemotherapies have been tried with limited outcome. We report a case of ITP that was unresponsive to multiple agents including high-dose steroid, IVIG, Rho(D) immune globulin, rituximab, cyclosporine, azathioprine, vincristine, mycophenolate mofetil, romiplostim, and eltrombopag; however, it achieved complete remission with combination treatment of cyclophosphamide and eltrombopag.

## 1. Introduction

Idiopathic thrombocytopenic purpura or immune thrombocytopenic purpura (ITP) is a chronic disorder with thrombocytopenia that affects approximately 1 in 10,000 people. Primary ITP is a heterogeneous disease and underlying mechanisms include platelet autoantibody (detected only in 60% of cases [1]) directed against glycoproteins in the platelet membrane, activation of T cells, tolerance loss in T and B cells, and the development of cytotoxic T cells [2]. However, the initial inciting event in ITP is still unknown. In rare cases, morphologic alteration of megakaryocytes in the bone marrow can be observed, supporting the hypothesis that ITP may result from disruptions of megakaryocytopoiesis and thrombopoiesis [3].

ITP is often a diagnosis of exclusion and the differential diagnosis among other causes includes pseudothrombocytopenia, drug induced thrombocytopenia, microangiopathic thrombocytopenia, bone marrow failure, and congenital thrombocytopenia. The primary (idiopathic) form of ITP is to be distinguished from secondary ITP (associated with other diseases, such as systemic lupus erythematosus, chronic lymphocytic leukemia, lymphoma, HIV/AIDS, and hepatitis C). In adults, chronic disease is defined as disease persisting for more than 6 months. Chronic refractory ITP may be defined as the failure of any modality to keep the platelet count above 20 × 10^9^/L for an appreciable time without unacceptable toxicity.

High-dose cyclophosphamide has been described as effective therapy for ITP [4] and we describe a unique case treated with high-dose cyclophosphamide and eltrombopag, which resulted in a long-lasting complete remission (CR).

## 2. Case Presentation

**Figure 1:**
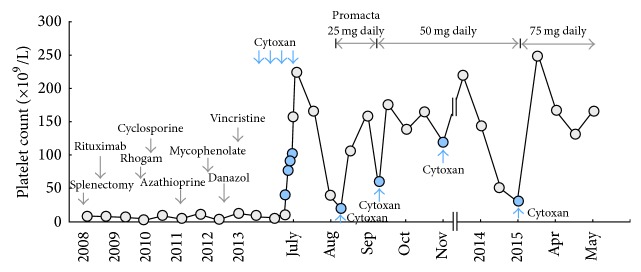
Platelet response to sequential treatments for ITP.

A 43-year-old female was diagnosed with ITP 6 years ago during pregnancy (male fetus) and had been treated with steroids. Over the years, she underwent a number of treatments for “refractory” ITP including, in addition to corticosteroids, splenectomy, IVIG, Rho(D) immune globulin, rituximab, cyclosporine, azathioprine, vincristine, mycophenolate mofetil, romiplostim, and eltrombopag (Figure 1). She responded to romiplostim; however, she developed peripheral neuropathy, which is one of the common adverse effects of romiplostim, and treatment was halted. She subsequently was treated with eltrombopag combined with danazol; however, the regimen was stopped because of intolerance even though her platelets improved on this regimen. She was being treated according to the regimen proposed by Arnold et al. [5] but did not achieve a good response. She experienced a left occipital hemorrhage when her platelet count was less than 5 × 10^9^/L. She underwent 12 days of plasmapheresis with no improvement and was transferred to our center.

Upon transfer, a marrow examination showed normal cellularity with megakaryocyte hyperplasia, multilineage hematopoiesis, and no dysplasia. She received high-dose cyclophosphamide (50 mg/kg/day) for 4 days for the first cycle, followed by four more cycles of 500 mg cyclophosphamide IV 4–6 weeks apart based on count recovery. Eltrombopag (25 mg daily) was added with cycle 2 of cyclophosphamide and dose was increased up to 50 mg and 75 mg after cycle 3 and cycle 5 of cyclophosphamide, respectively. She is currently on eltrombopag 75 mg daily and maintaining normal platelet counts (Figure 1). Except neutrophils (ANC) decline to 0.96 × 10^9^/L after 1st cycle of high-dose cyclophosphamide, she has had no significant alterations of ANC and platelets nor has she had any clinical evidence of infections.

## 3. Discussion

Various therapeutic modalities have been shown to be effective in ITP [6, 7]. Splenectomy (complete response (CR) rate 70%) works by reducing the clearance of autoantibody-coated platelets [8], intravenous (IV) anti-D and IV immunoglobulin (IVIG) cause extravascular hemolysis of anti-D or autoantibody-sensitized red blood cells (RBCs) by splenic macrophages, which results in decreased splenic sequestration of autoantibody-sensitized platelets [9] and an increase in platelet counts, and rituximab [10] depletes B cells; intravenous immunoglobulin also contains anti-idiotypic antibodies and decreases autoantibody production but the exact mechanism is still not clear [11]. Corticosteroids suppress lymphocytes and macrophages (via apoptosis) and reduce the destruction of platelets [12]. Thrombopoietin and thrombopoietic agents stimulate megakaryocyte progenitors and increase production of platelets [13].

Immunosuppression with agents such as azathioprine [14] (via blockade of the pathway for purine synthesis), cyclosporine [15] (by decreased production and release of IL-2 and IL-2-induced activation of resting T lymphocytes), and mycophenolate mofetil (by inhibiting inosine monophosphate dehydrogenase which inhibits de novo guanosine nucleotide synthesis pathways that are required for T and B lymphocytes) results in suppression of cell and antibody mediated immunity. Platelet transfusions provide transient replacement for urgent and selective medical situations. Other selective antibodies (anti-CD44, anti-CD154) have shown activity for ITP therapy [16, 17] as well. Most of ITP patients undergo treatments with corticosteroids, and if there is no lasting response, splenectomy is performed. 60–70% of patients with ITP achieve stable responses with this strategy, defined as platelet counts >30 × 10^9^/L or not requiring additional therapy for symptomatic thrombocytopenia.

A rare patient will undergo hematopoietic stem cell transplantation for refractory ITP [18–20]. High-dose (HD) cyclophosphamide given over multiple days as used for conditioning before allogeneic stem cell transplantation [21, 22] and for graft versus host disease prophylaxis [23] is profoundly immunosuppressive. It has also shown activity in the treatment of aplastic anemia [24], severe autoimmune hemolytic anemia [25], and other autoimmune diseases [26]. HD cyclophosphamide has also been described for the treatment of refractory ITP [27]. Eltrombopag is an FDA approved thrombopoietin receptor agonist for the treatment of patients with chronic ITP [28, 29]. Standard treatment with single agent glucocorticoid therapy and immunosuppressive agents is effective to induce remissions in patients with chronic refractory ITP [30]. Combining various agents has shown good activity in patients with refractory chronic ITP.

In the present case pulsed HD cyclophosphamide in combination with a thrombopoiesis stimulating agent led to a long-lasting remission of ITP without causing neutropenia or infections. Our patient continues to have a stable range of platelets with more than 12 months of follow-up. Thus, while the contributions of cyclophosphamide and eltrombopag, respectively, cannot be differentiated in the present case, eltrombopag was well tolerated in combination with HD cyclophosphamide in contrast to the intolerance observed in an earlier treatment attempt with danazol. Cyclophosphamide may modify the immunological milieu, resulting in an enhanced response to the thrombopoietin receptor agonist.
